# A systems-approach reveals human nestin is an endothelial-enriched, angiogenesis-independent intermediate filament protein

**DOI:** 10.1038/s41598-018-32859-4

**Published:** 2018-10-02

**Authors:** Philip Dusart, Linn Fagerberg, Ljubica Perisic, Mete Civelek, Eike Struck, Ulf Hedin, Mathias Uhlén, David-Alexandre Trégouët, Thomas Renné, Jacob Odeberg, Lynn M. Butler

**Affiliations:** 1 Science for Life Laboratory, School of Biotechnology, Kungliga Tekniska Högskolan (KTH) Royal Institute of Technology, SE-171 21 Stockholm, Sweden; 2 Clinical Chemistry and Blood Coagulation, Department of Molecular Medicine and Surgery, Karolinska Institute, SE-171 76 Stockholm, Sweden; 3 Vascular Surgery, Department of Molecular Medicine and Surgery, Karolinska Institute, SE-171 76 Stockholm, Sweden; 4 Department of Biomedical Engineering, University of Virginia, Charlottesville, USA; 5 Sorbonne Universités, UPMC Univ Paris 06, UMR_S 1166, Team Genomics & Pathophysiology of Cardiovascular Diseases, Paris, France; 6 ICAN Institute for Cardiometabolism and Nutrition, Paris, France; 7 Institute for Clinical Chemistry and Laboratory Medicine, University Medical Centre Hamburg-Eppendorf, D-20246 Hamburg, Germany; 8 Coagulation Unit, Centre for Hematology, Karolinska University Hospital, SE-171 76 Stockholm, Sweden

## Abstract

The intermediate filament protein nestin is expressed during embryonic development, but considered largely restricted to areas of regeneration in the adult. Here, we perform a body-wide transcriptome and protein-profiling analysis to reveal that nestin is constitutively, and highly-selectively, expressed in adult human endothelial cells (EC), independent of proliferative status. Correspondingly, we demonstrate that it is not a marker for tumour EC in multiple malignancy types. Imaging of EC from different vascular beds reveals nestin subcellular distribution is shear-modulated. siRNA inhibition of nestin increases EC proliferation, and nestin expression is reduced in atherosclerotic plaque neovessels. eQTL analysis reveals an association between SNPs linked to cardiovascular disease and reduced aortic EC nestin mRNA expression. Our study challenges the dogma that nestin is a marker of proliferation, and provides insight into its regulation and function in EC. Furthermore, our systems-based approach can be applied to investigate body-wide expression profiles of any candidate protein.

## Introduction

Nestin is a type-IV intermediate filament (IF), first identified in the neuroepithelial stem cells of the embryonic rat neural tube^1^. Since then it has been widely described as a marker for stem or progenitor cells, mainly in the developing central nervous system^2,3^, but also heart^4,5^, bone marrow^6^, and others. Following cell differentiation, nestin is reportedly replaced by other cell-type specific IF^7–9^, and its expression is predominantly restricted to areas of regeneration in healthy adult tissues^3^, such as skeletal muscle^10^, hair follicles^11^, dopaminergic neurons^12^ and neural stem cells^13,14^. Nestin is also expressed in kidney podocytes^15,16^ and the neuromuscular junction^17^. In the adult cardiovascular system, nestin is expressed in actively proliferating endothelial cells (EC), but is considered absent from mature vasculature^6,18,19^. Thus, it has been suggested as a potential therapeutic target for tumour-associated angiogenesis^18,20–23^ and a prognostic marker^24–26^.

There are few studies of nestin expression in humans, with current knowledge almost entirely extrapolated from observations in animal models. Here, we perform a transcriptome and antibody-based profiling analysis of 37 human organs, which reveals body-wide EC-enriched nestin expression, independent of proliferative status. In addition, we analyse different tumour types to reveal that nestin is not a specific marker of tumour-associated EC, and its expression level is not an independent prognostic factor. We use primary human EC from four vascular beds to examine nestin spatial profiles under static and flow culture, and demonstrate an unexpected role for nestin in the inhibition of EC proliferation. Correspondingly, we show reduced nestin expression in atherosclerotic plaque neovessels.

Our study identifies nestin as a tissue-wide, EC-enriched IF, challenging its validity as a stem/progenitor or proliferative cell marker, its differential regulation between cell types and species, and its functional role in the adult vasculature.

## Results

### Nestin is an endothelial-enriched protein in healthy human adult tissues

**Figure 1:**
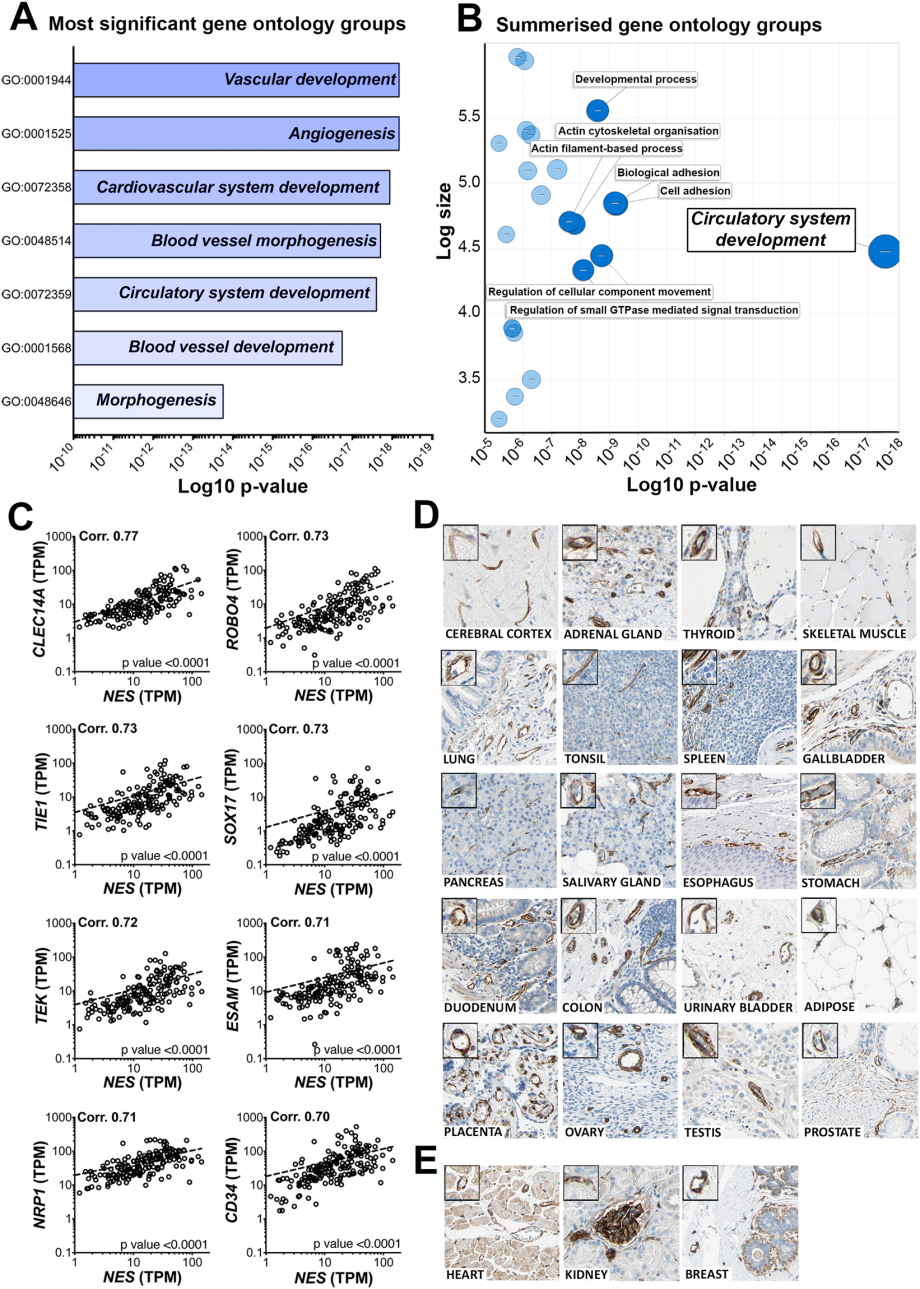
Nestin is a pan-EC enriched protein in the human adult. RNA-seq data from 176 individual samples from 37 different human tissues were used to generate Spearman pair wise correlation values between *NES* transcript expression and transcripts for all other mapped protein coding genes. Gene ontology (GO) analysis was performed on the 150 transcripts that were most highly correlated with *NES*: (**A**) the 10 most significant GO functional groupings and (**B**) a summary of GO functional groupings with removal of redundant terms (plot produced using REVIGO [http://revigo.irb.hr/] before modification). (**C**) Correlation plots showing the relationship between *NES* and the EC-enriched transcripts *CLEC14A*, *ROBO4*, *TIE1*, *SOX17*, *TEK*, *ESAM*, *NRP1* and *CD34*. Correlation values and corresponding p-values are shown in the top left and bottom right of each scatter plot, respectively. Human tissue sections from (**D**) cerebral cortex, adrenal gland, thyroid, skeletal muscle, lung, tonsil, spleen, gallbladder, pancreas, salivary gland, esophagus, stomach, duodenum, colon, urinary bladder, adipose, placenta, ovary, testis, prostate and (**E**) heart, kidney and breast were stained for protein encoded by *NES*. See also Supplemental Fig. 1 and Supplemental Tables 1 and 2.

We performed RNAseq tissue transcript profiling of 176 samples collected from 37 adult human organs (Table S1) as part of the Human Protein Atlas Project, version 17 (HPA; www.proteinatlas.org)^27^. Transcripts per kilobase million (TPM) values were calculated for 22,130 mapped protein-coding genes. We generated Spearman pair wise correlation values between nestin transcripts (*NES*) and transcripts for all other mapped protein coding genes (‘test’ transcripts); high correlation values with known cell-type enriched transcripts could indicate corresponding enrichment of nestin in that cell type. Correlations were observed between *NES* and test transcripts (corr. >0.65 n = 234, all p-value < 0.0001 [>0.70 n = 92]). Gene ontology (GO) analysis^28^ (http://geneontology.org/) was performed on the 150 test transcripts that most highly correlated with *NES* (all corr. >0.67, p-value < 0.0001) (Table S2). The most significant over-represented groups were related to vascular or EC function (Fig. 1A). We used REViGO^29^ to summarise and remove redundant terms from all GO groupings identified (Table S2); ‘*circulatory system development*’ was the stand-alone most significant (p-value 10^18^) (Fig. 1B). Consistent with these functional groupings, of the 150 test transcripts that most highly correlated with *NES*, we had previously identified >50% as being highly EC-enriched across human organs^30^, e.g. *CLEC14A*, *ROBO4*, *TIE1*, *SOX17*, *TEK*, *ESAM*, *NRP1* and *CD34 (*corr. 0.77, 0.73, 0.73, 0.73, 0.72, 0.71, 0.71 and 0.70, respectively, all p-value < 0.0001) (Fig. 1C). TPM levels were of comparable magnitude between these transcripts (mean TPM, all tissues ± SD: *NES* 22.1 ± 23.6*, CLEC14A* 15.9 ± 20.0, *ROBO4* 11.8 ± 18.4, *TIE1* 13.5 ± 18.4, *SOX17* 4.9 ± 8.9, *TEK* 13.6 ± 17.5, *ESAM* 29.6 ± 43.7, *NRP1* 52.5 ± 45.7, *CD34* 54.8 ± 72.9). We replicated this analysis in an independent sample set, using RNAseq data from the Genotype-Tissue Project (GTEx) portal (www gtexportal.org)^31^, from 25 human organs (2841 samples) (Table S1). *NES* transcript TPM values strongly correlated with TPM values for the same panel of EC-enriched transcripts: *CLEC14A*, *ROBO4*, *TIE1*, *SOX17*, *TEK*, *ESAM*, *NRP1* and *CD34* (corr. 0.78, 0.77, 0.78, 0.76, 0.72, 0.70, 0.72 and 0.73 respectively, all p-value < 0.0001) (Fig. S1A–F), in this replication dataset. This analysis indicated that *NES* transcript expression is enriched in EC across organs, and we used protein profiling to confirm that nestin protein was specifically expressed in the EC compartment of the human adult cerebral cortex, adrenal gland, thyroid, skeletal muscle, lung, tonsil, spleen, pancreas, salivary gland, esophagus, stomach, duodenum, colon, urinary bladder, adipose, placenta, ovary, testis and prostate (Fig. 1D). Nestin was also expressed in EC of the heart, kidney, and breast, although in these tissues, consistent with previous reports^32–34^, non-EC expression was observed in cardiomyocytes, renal glomeruli, and myoepithelial cells, respectively (Fig. 1E).

### EC proliferation is not a pre-requisite for nestin expression *in vivo*

**Figure 2:**
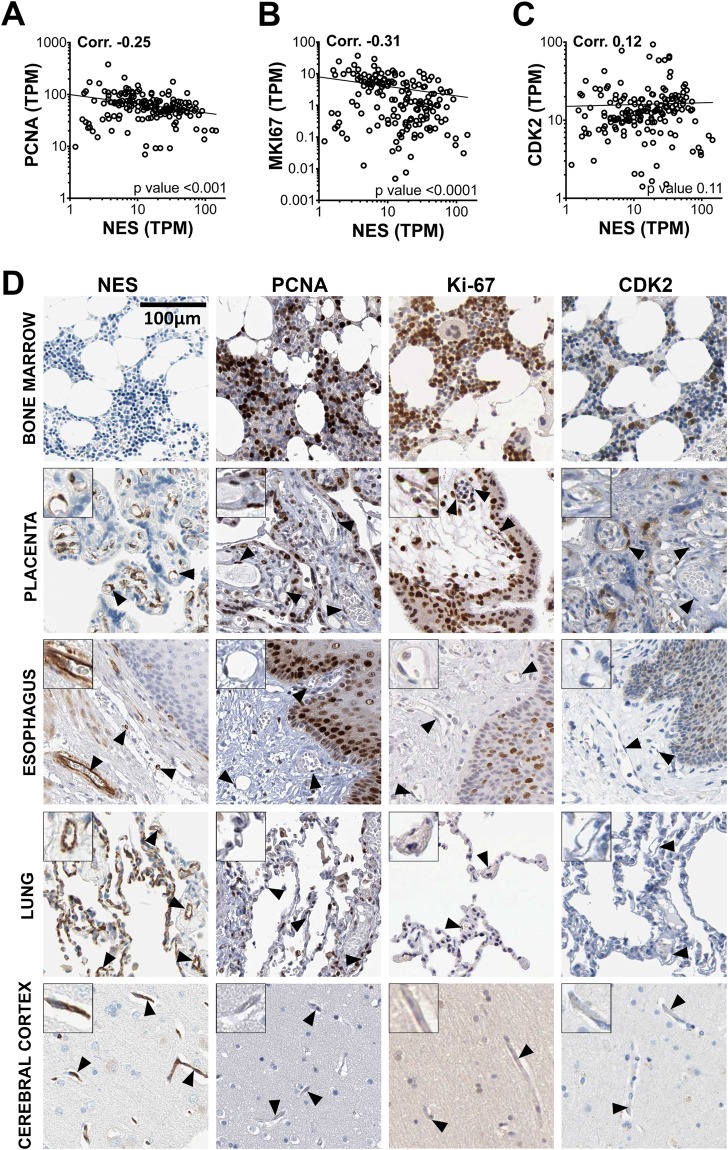
Nestin expression is not restricted to proliferating EC. RNA-seq data from 176 individual samples from 37 different human tissues were used to generate Spearman pair wise correlation values between *NES* transcript values and those encoding for the known proliferation markers (**A**) proliferating cell nuclear antigen (*PCNA*) (**B**) marker of proliferation Ki-67 (*MKI67*) and (**C**) cyclin-dependent kinase 2 (*CDK2*). Correlation values and corresponding p-values are shown in the top left and bottom right of each scatter plot, respectively. (**D**) Human tissue sections from the bone marrow, placenta, esophagus, lung and cerebral cortex were stained for protein encoded by *NES*, *PCNA*, *MKI67, and CDK2*. See also Supplemental Fig. 1 and Supplemental Table 1.

The vast majority of the adult human vasculature is quiescent^35^, but as nestin expression is currently considered largely restricted to progenitor or proliferating cells, we investigated the proliferative status of nestin-positive EC. In the HPA dataset, *NES* transcript TPM values did not positively correlate with those for the proliferation markers *PCNA* (proliferating cell nuclear antigen), *MKI67* (antigen KI-67) or *CDK2* (cyclin-dependent kinase 2), which are expressed in replicating EC^36–38^ (corr. −0.25, −0.31 and 0.12, respectively) (Fig. 2A–C). PCNA, KI67 and CDK2 protein was expressed in the highly proliferative hematopoietic cells of the bone marrow (Fig. 2D, row 1), occasional EC in the placenta (Fig. 2D, row 2) and squamous epithelial cells of the esophagus (Fig. 2D, row 3), but not EC of the esophagus, lung, cerebral cortex (Fig. 2D, row 3, 4, 5), or other tissues, including adrenal gland, thyroid, skeletal muscle, tonsil, spleen, pancreas, salivary gland, stomach, duodenum, colon, urinary bladder, adipose, ovary and prostate (images not shown). As nestin was expressed in the EC of all these tissues, its expression is therefore not dependent on proliferative status.

### Nestin expression is not specific to tumour EC

Nestin has been described as a specific marker for vessels in various tumour types^22,39,40^, with prognostic potential^25,41–43^. Here, we used transcriptomic and protein profiling of five tumour types to determine if EC nestin expression is modified in malignant tissue, and to determine its value as a prognostic marker for survival.

RNAseq data for *NES* and the EC transcripts, *PECAM1* (*CD31*) and *CD34*, in kidney renal clear cell carcinoma (KIRC), bladder urothelial carcinoma (BLCA), lung adenocarcinoma (LUAD), stomach adenocarcinoma (STAD), glioblastoma multiforme (GBM) and corresponding normal tissue, was downloaded from the Cancer Genome Atlas (TCGA) and Genotype-Tissue Expression (GTEx) project. Protein profiling and survival analysis was performed as part of our HPA pathology atlas^44^.

**Figure 3:**
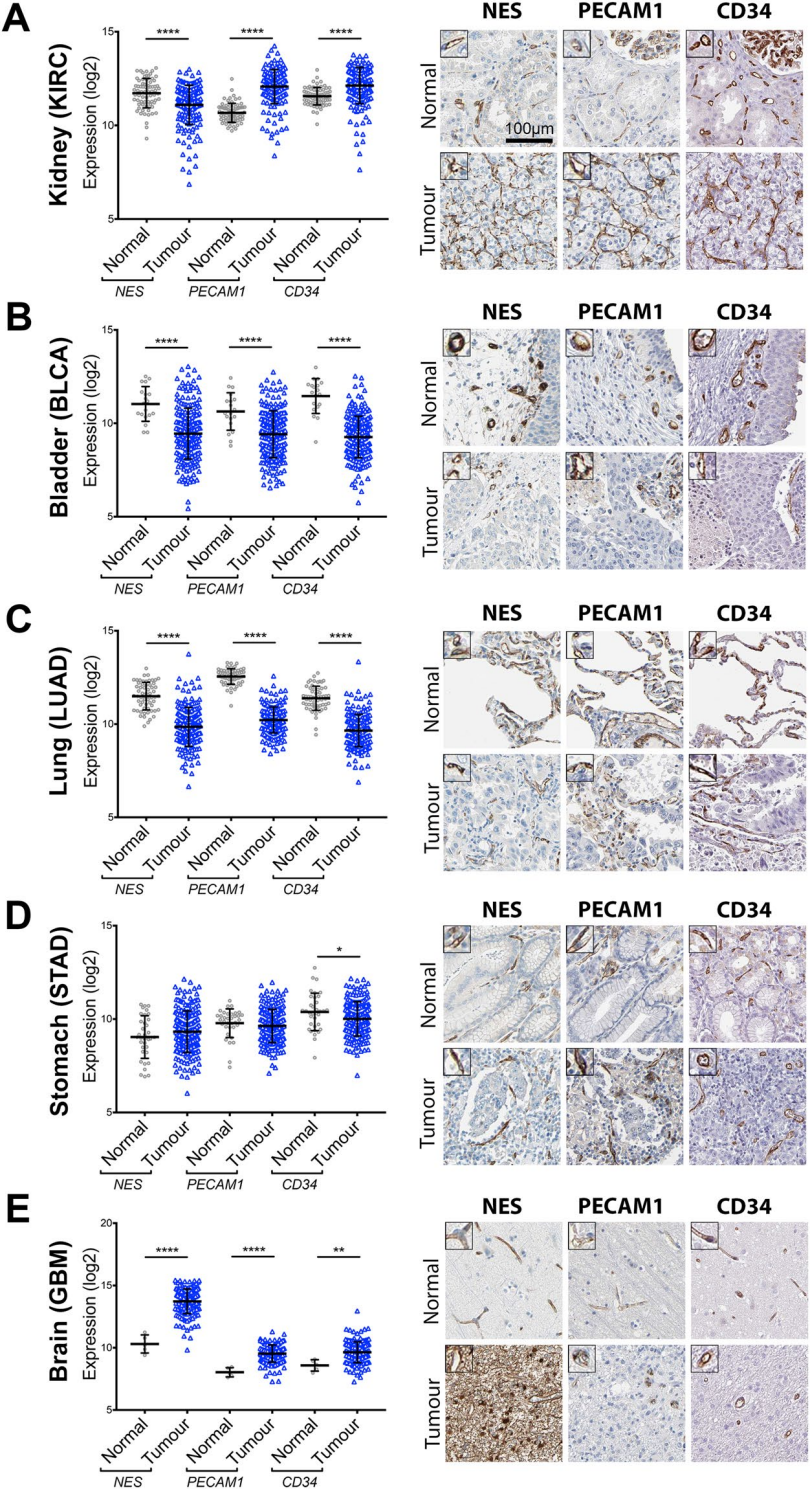
Nestin expression is not specific to tumour EC. RNA-seq data from the TCGA consortium for *NES*, *PECAM*1 and *CD34* expression ±SD (graphs on left), and corresponding IHC images of tissue sections stained for the encoded proteins, in (**A**) kidney renal clear cell carcinoma (KIRC), (**B**) bladder urothelial carcinoma (BLCA), (**C**) lung adenocarcinoma (LUAD), (**D**) stomach adenocarcinoma (STAD), (**E)** glioblastoma multiforme (GBM) and the corresponding normal tissue. Unpaired *t-test* *p-value < 0.05, **<0.01 ****<0.0001. See also Supplemental Fig. 2.

*CD34* and *PECAM1* levels were elevated in KIRC vs. normal kidney (mean Log2 KIRC vs. normal kidney: *CD34* 12.1 vs. 11.5, *PECAM* 12.1 vs. 10.7, both p-value < 0.0001) (Fig. 3A), consistent with reports that KIRC is highly-vascularised^45^. In contrast, *NES* was decreased (mean Log2 KIRC vs. normal kidney: *NES* 11.1 vs. 11.7 vs., p-value < 0.0001). EC-enriched protein expression of nestin, CD34 and PECAM1 was observed in both normal kidney and KIRC (Fig. 3A, right image panels). There was an increase in vascular density in KIRC, and the pattern and extent of EC staining was comparable for nestin, CD34 and PECAM1. The reduction in *NES* mRNA in KIRC vs. normal tissue, despite the increase in nestin-positive vessels, could be due to loss of glomerular-associated *NES* (see Fig. 1A). *NES* expression was associated with poor outcome in renal cancer (Fig. S2A), as were *CD34* and *PECAM1*.

*NES*, *PECAM1* and *CD34* mRNA expression was lower in BLCA vs. normal bladder (mean Log2 BLCA vs. normal bladder: *NES* 9.4 vs. 11.0, *PECAM* 9.4 vs. 10.6, CD34 9.3 vs. 11.5, all p-value < 0.0001) and in LUAD vs. normal lung (mean Log2 LUAD vs. normal lung: *NES* 9.8 vs. 11.5, *PECAM* 10.2 vs. 12.5, CD34 9.6 vs. 11.4, all p-value < 0.0001) (Fig. 3B,C, respectively). EC-enriched protein expression of nestin, CD34 and PECAM1 was observed in BLCA, LUAD and corresponding normal tissue (Fig. 3B,C, right image panels). Consistent with the RNAseq, vascular density was lower in BLCA and LUAD vs. normal tissues.

*NES* and *PECAM1* mRNA expression was unchanged in STAD vs. normal stomach, but *CD34* was moderately reduced (Fig. 3D). Protein profiling confirmed EC-enriched expression of all three transcripts, and a comparable degree of EC staining in both normal stomach and STAD (Fig. 3D, right image panels).

*NES* mRNA expression was highly elevated in GBM vs. normal brain (mean Log2 GBM vs. normal brain: *NES* 13.7 vs. 10.3, p-value < 0.0001). *PECAM1* and *CD34* mRNA were also elevated in GBM vs. normal brain, but to a lesser extent (Fig. 3E). Protein profiling confirmed increased nestin staining in GBM, compared to normal brain, but this expression was not restricted to EC. CD34 and PECAM1 protein staining were EC-enriched in normal brain and GBM (Fig. 3E, right image panels). *NES*, *CD34* nor *PECAM1* expression was associated with prognosis in urothelial cancer, lung cancer, stomach cancer or glioma (Fig. S2B–E).

Taken together, these data show that nestin is a marker of EC in both normal and tumour tissues (with the exception of GBM), and thus is not specifically expressed by tumour-associated EC.

### EC nestin distribution is modulated by laminar flow *in vitro*

**Figure 4:**
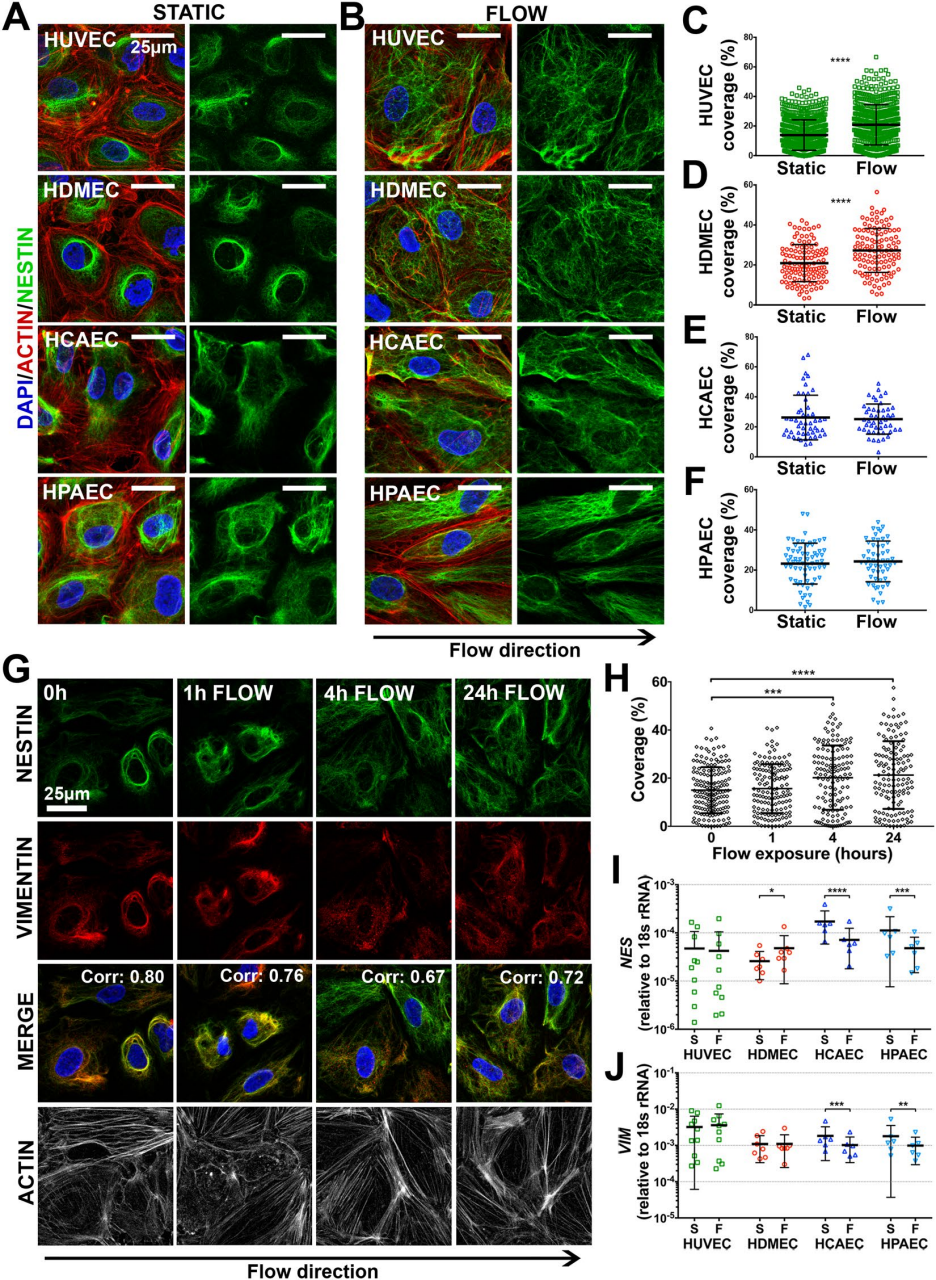
EC nestin is regulated by laminar shear stress *in vitro*. Immunofluorescence staining of nestin in HUVEC, HDMEC, HCAEC and HPAEC cultured under (**A**) static conditions, or (**B**) 10 dyne/cm^2^ laminar shear stress for 24 hours. Quantification of nestin spatial distribution in (**C**) HUVEC, (**D**) HDMEC, (**E**) HCAEC and (**F**) HPAEC. Each point represents an individual cell (n = 3–19 different experiments) Unpaired *t-test* *p-value < 0.05, **<0.01 ****<0.0001. (**G**) HUVEC were cultured under 10 dyne/cm^2^ laminar shear stress for 0 h, 1 h, 4 h or 24 h, and stained for nestin, vimentin, actin, and DAPI. Pearson’s co-localisation value for nestin and vimentin is displayed on the top right of the merged images. (**H**) Quantification of nestin spatial distribution over the time course (n = 6); significance was calculated using one-way ANOVA. (**I**) *NES* and (**J**) *VIM* mRNA expression in EC under both static conditions and laminar shear stress, displayed relative to 18 s rRNA (n = 6–10). All graphs show means ± SD.

Nestin transcription and sub-cellular protein distribution was characterised in human EC from four different vascular beds. EC from umbilical vein (HUVEC), dermal microvessels (HDMEC), coronary artery (HCAEC), and pulmonary artery (HPAEC) were cultured *in vitro* under static conditions, or laminar shear stress (10 dyne/cm^2^). Under static conditions, EC grew in the characteristic cobblestone pattern and nestin was localised in a cytoplasmic perinuclear pattern around the nuclear membrane in all EC types, with some wider cytoplasmic expression (Fig. 4A). Exposure to laminar shear stress caused the EC to elongate in shape and induced the formation of a highly filamentous network in all EC (Fig. 4B), with a corresponding increase in spatial distribution in HUVEC and HDMEC (mean cell coverage, static vs flow (%) 13.9 ± 0.4 vs. 20.8 ± 0.6, and 20.9 ± 0.9 vs. 27.3 ± 1.0, respectively, p-values < 0.0001) (Fig. 4C,D). A time-course assay revealed shear stress-induced nestin spatial reorganisation was induced by 4 hours of flow exposure (Fig. 4G) (mean cell coverage (%) at 0 h: 15.0% ± 0.7 vs. 4 h: 20.2 ± 1.1, p-value 0.0004), with no further change observed after 24 h of flow exposure (mean cell coverage (%) 21.3 ± 1.2) (Fig. 4H). To determine the relative contribution of *de novo NES* transcription vs. redistribution of existing protein, *NES* mRNA expression was measured by qPCR. Consistent with the protein staining, under static conditions *NES* mRNA was expressed at the highest levels in arterial EC (HCAEC and HPAEC) (Fig. 4I). Upon exposure to laminar shear stress (24 h) *NES* was reduced in HCAEC and HPAEC (fold change 0.40 ± 0.02, and 0.47 ± 0.03, respectively, p-values < 0.0001), increased in HDMEC (fold change 1.91 ± 0.3, p-value 0.02) and was unchanged in HUVEC (Fig. 4I). Thus, exposure to shear stress had no consistent effect between EC types on *NES* mRNA expression, and so the changes in nestin distribution were not dependent on *de novo NES* mRNA transcription.

### EC nestin co-localises with vimentin

Nestin has a short N-terminal head domain that inhibits self-assembly, but it can form heterodimers with other IF proteins^46–48^. Vimentin (VIM) is the principle IF in EC^49,50^ and can co-assemble with nestin in other cell types^8,46,47,51^ therefore, we investigated the relationship between nestin and vimentin in EC.

*VIM* mRNA was expressed at higher levels than *NES* mRNA in all EC types (mean fold difference *VIM* vs. *NES*: HUVEC 77.9 ± 33.2, HDMEC 45.3 ± 25.4, HCAEC 10.2 ± 1.4, HPAEC 16.3 ± 3.1) (Fig. 4I vs. J). Exposure to laminar shear stress downregulated *VIM* mRNA in HCAEC and HPAEC (fold change 0.59 ± 0.04, p-value < 0.0001, and 0.64 ± 0.06, p-value 0.003, respectively), as was observed for *NES*. Expression in HUVEC and HDMEC was unchanged (Fig. 4J). Like nestin, vimentin protein was localised around the nuclear membrane under static conditions and underwent shear stress induced sub-cellular redistribution. Vimentin and nestin showed a high degree of subcellular co-localisation under static and flow exposed conditions (Fig. 4G) (Corr.: 0 h: 0.80 ± 0.02, 1 h: 0.76 ± 0.04, 4 h: 0.67 ± 0.03, 24 h: 0.72 ± 0.03). No correlation existed between vimentin and the cytoskeletal protein actin (Corr. all <0.05). These data indicate that common regulatory processes underlie the spatial distribution of EC nestin and VIM under static and laminar shear stress.

### EC proliferation is not a pre-requisite for nestin expression *in vitro*

**Figure 5:**
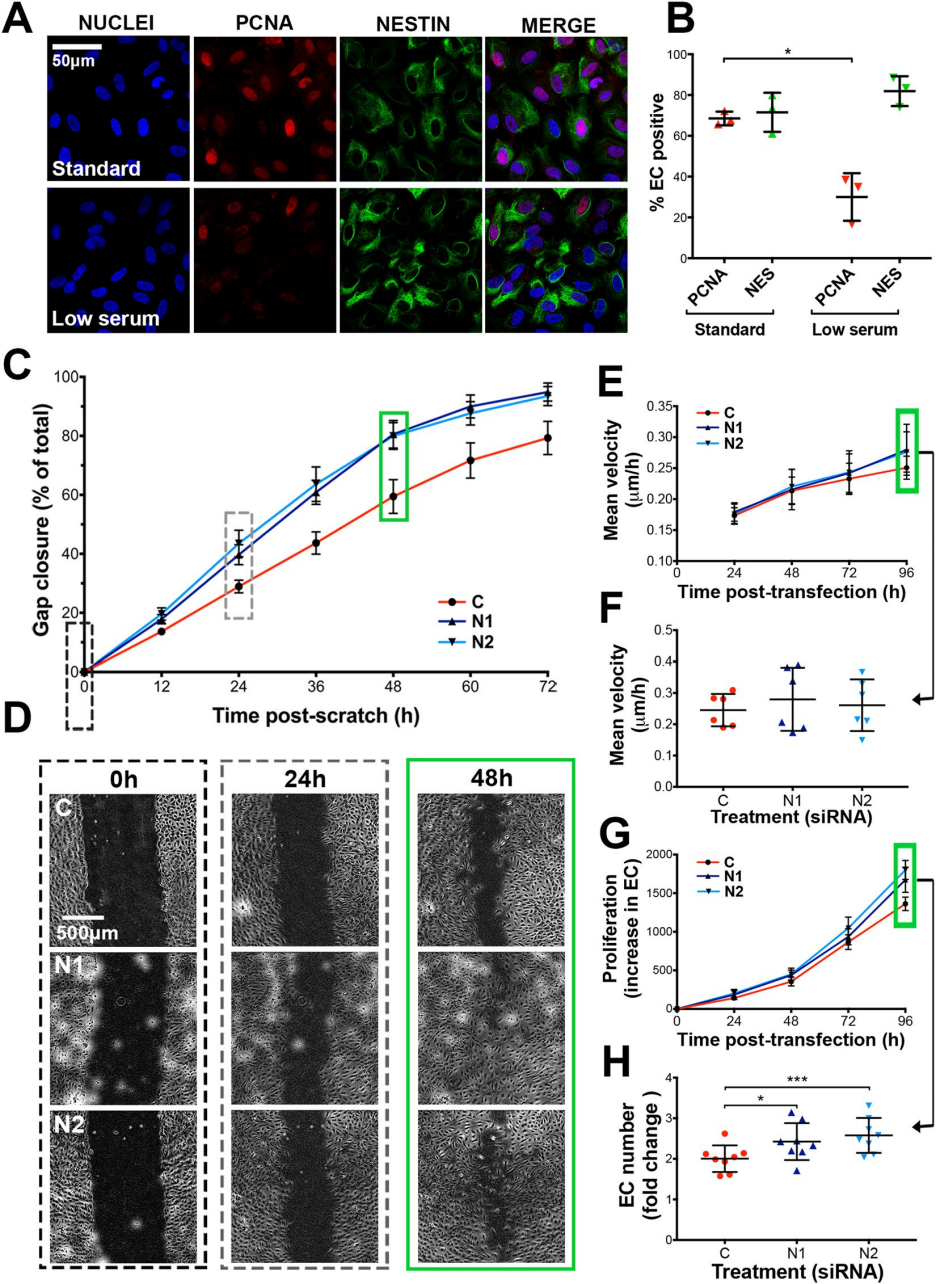
Nestin siRNA knockdown increases EC proliferation *in vitro*. (**A**) Immunofluorescence staining of nestin and PCNA in HUVEC cultured in standard or low serum medium (**B**) Quantification of nestin or PCNA positive cells (n = 3, 34–73 cells analysed/experiment) Paired *t-test* *p-value < 0.05. (**C**) HUVEC were treated with control (‘C’) or one of 2 anti-nestin (‘N1’ and ‘N2’) siRNAs and cultured to confluence for 48 h, before a ‘wound’ was generated and gap closure was measured at 12 h intervals (means ± SEM, n = 13). (**D**) Representative phase contrast images were captured at 0 h, 24 h and 48 h post wound creation (48 h, 72 h and 96 h post-transfection, respectively). HUVEC were seeded to 50% confluence and transfected with C, N1 or N2 siRNA, subsequently (**E**) migration velocity (n = 6), and (**G**) cell proliferation (n = 8) were measured every 24 h for 96 h. Individual data points from 96 h post-transfection are shown for velocity and migration (**F** and **H**, respectively). Green boxes indicate measurements taken at the same time point post-transfection (96 h). (All graphs: mean ± SD, significance calculated by one-way ANOVA).

We demonstrated that EC nestin expression *in vivo* is not restricted to cells that express markers of replication (Fig. 2). We used serum starvation to inhibit EC division *in vitro*^52^ to further investigate the relationship between nestin and proliferative status. EC cultured in low serum medium had reduced PCNA positivity (positive cells (mean %) ±SEM: 30.0 ± 6.7 vs. 68.5 ± 1.9, low serum vs. standard, respectively, P-value 0.02). However, the number of nestin positive EC was not affected (positive cells (mean %) ±SEM: 81.9 ± 4.2 vs. 71.5 ± 5.5, low vs. standard serum, respectively) (Fig. 5A,B). This data further supports that proliferation is not a pre-requisite for EC nestin expression.

### Nestin knockdown increases EC proliferation

To further examine EC nestin function, EC were transfected with 1 of 2 different siRNA sequences targeting *NES* (‘N1’ and ‘N2’) or a control scrambled siRNA sequence (‘C’). Inhibition of *NES* expression was confirmed by qPCR (inhibition (%) 75 ± 13 and 82 ± 8, N1 and N2, respectively), Western blot, and immunofluorescence staining (Fig. S3). Nestin inhibition did not affect the expression or subcellular organisation of vimentin under either static or flow conditions (Fig. S4). EC were cultured to confluence, before a ‘wound’ was created in the monolayer and subsequent ‘closure’ monitored. EC transfected with siRNAs targetting *NES* had significantly faster gap closing compared to the control (Fig. 5C,D) (gap closure after 48 h ±SEM: 80.6% ± 4.7 and 79.9% ± 4.6, N1 and N2 respectively p-values 0.0025 and 0.0004, compared to 59.4% ± 5.7 for C) (Fig. 5C). This accelerated gap closing could be due to: (1) increased EC motility or (2) increased EC proliferation. To determine the relative contribution of each, the position and number of individual EC were recorded over a period of 96 hours following transfection (N1, N2 and C siRNA). EC migration velocity was not affected by N1 or N2 siRNA (Fig. 5E,F), however cell proliferation was significantly increased in N1 and N2 treated EC (fold increase in EC number over 96 h post-transfection ±SEM: 2.4 ± 0.16 and 2.6 ± 0.15, N1 and N2 respectively, p-values 0.011, 0.0002, compared to 2.0 ± 0.12 for C) (Fig. 5G,H). Taken together, these results indicate that inhibition of nestin increases cellular proliferation and thus, nestin expression inhibits the capacity for EC division.

### Nestin EC expression is lower in atherosclerotic plaques than in normal artery

**Figure 6:**
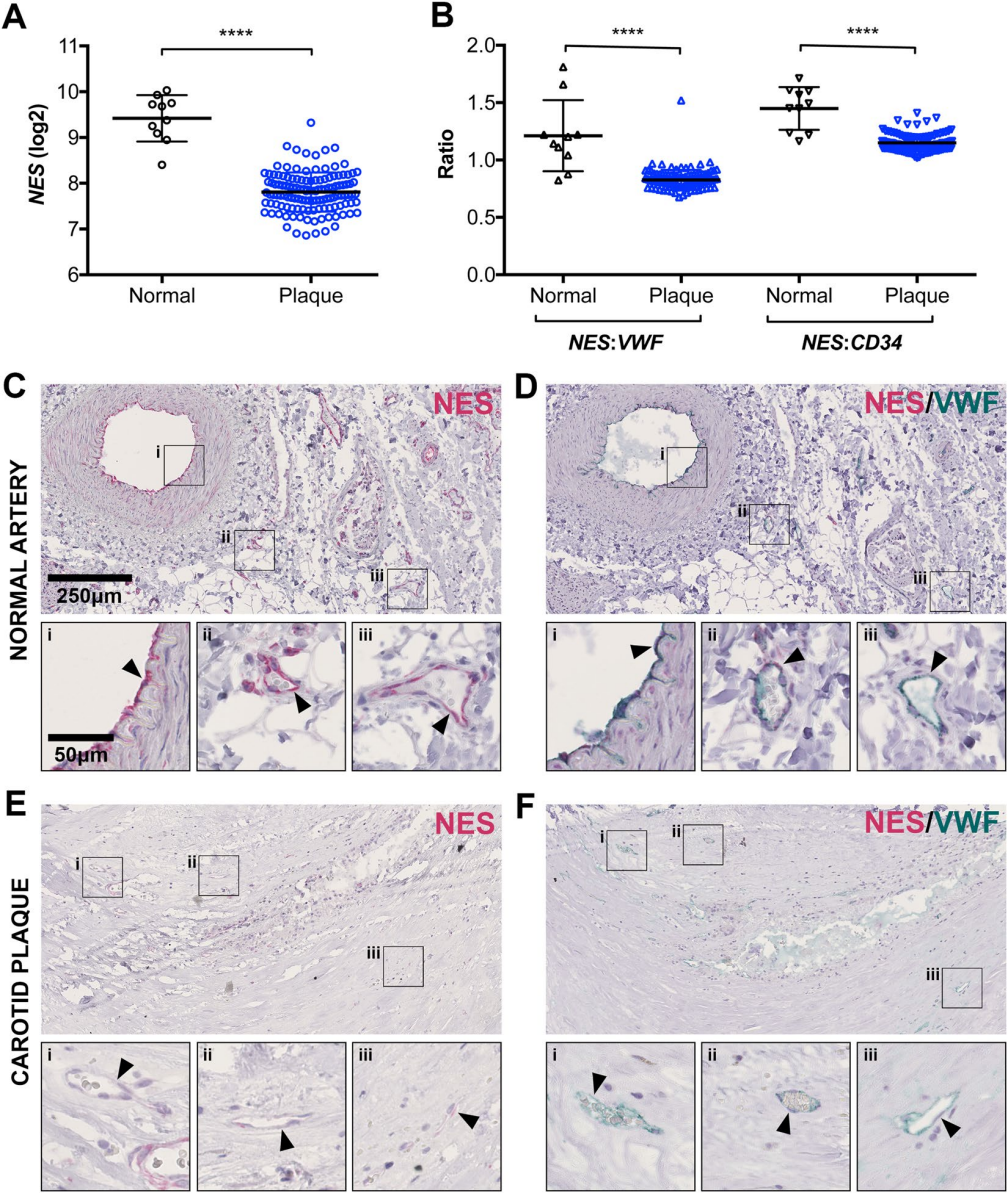
Nestin expression is lower in atherosclerotic plaque vs. normal artery. mRNA was prepared from 127 human carotid atherosclerotic plaques and 10 normal arteries and analysed by microarray to determine: (**A**) total *NES* mRNA expression and (**B**) the ratio of *NES* mRNA relative to the EC markers *VWF* or *CD34*. Unpaired *t-test* ****p-value < 0.0001. Sections of (**C**,**D**) normal artery or (**E**,**F**) carotid plaque were stained by immunohistochemistry for (**C**,**E**) NES or (**D**,**F**) both NES and VWF protein expression. Arrows highlight blood vessel EC.

The cellular redistribution of nestin under flow and the effects of nestin knockdown on wound healing and proliferation led us to investigate nestin expression in the context of atherosclerosis. We used the patient cohort Biobank of Karolinska Endarterectomies (BIKE)^53^, to measure *NES* transcription by mRNA microarray analysis in carotid atherosclerotic plaques of patients undergoing surgery for high-grade (>50%) carotid stenosis (n = 127, 87 symptomatic, 40 asymptomatic) and normal arteries (n = 10). *NES* mRNA expression was significantly lower in atherosclerotic plaques compared to normal arteries (Fig. 6A), as was the ratio of *NES* to the EC marker transcripts *VWF* and *CD34* (Fig. 6B). IHC staining showed that nestin in normal arteries was expressed both in adventitial micro-vessels and luminal lining, and occasional smooth muscle cells (Fig. 6C). The neovessels of atherosclerotic plaques had weak nestin staining, or were negative altogether (Fig. 6E). EC were consistently positive for VWF in both normal and atherosclerotic vessels (Fig. 6D,F).

### Genetic variants in the *NES* gene locus linked to early onset coronary heart disease are associated with lower EC *NES* expression

Two single nucleotide polymorphisms (SNPs) in the *NES* gene (rs3748570 and rs11582300) were previously reported as associated with early onset coronary heart disease (CHD)^54^, however no data linking these SNPs to nestin expression (cell type, or degree of) has been reported. We tested for a cis-acting *NES* expression QTL (eQTL) using data obtained from cultured aortic ECs (HAEC) of 147 individuals (Table S3, sheet 1)^55^. In a univariate analysis, both rs3748570 and rs11582300 were associated with *NES* expression (p = 1.19 * 10^−3^ and 5.29 * 10^−3^ respectively). The strongest association was found for rs3935541 (p = 1.15 * 10^−4^), which is a SNP located approximately 50 kb upstream of the *NES* transcription start site. The rs3935541 is in strong LD with rs3748570 (r^2^ = 0.76, D′ = 0.96) and rs11582300 (r^2^ = 0.57, D′ = 0.90), according to the SNAP database (http://archive.broadinstitute.org/mpg/snap/index.php). We performed a haplotype analysis of these 3 SNPs on *NES* expression (Table S3, sheet 2) in the HAEC dataset. Four haplotypes were inferred from the 3 selected SNPs. Compared to the most frequent GAT haplotype, all other 3 haplotypes showed decreased *NES* mRNA expression. The greatest, and the only significant (p = 0.023), decrease was observed for the AGC haplotype, the only haplotype carrying the minor allele of rs3935541. Haplotype data were then compatible with the sole effect of the rs3935541 that explained ~4% of HAEC *NES* expression (p = 0,437 for rejecting this hypothesis).

## Discussion

Here, we used a systems-based approach, combining RNAseq analysis and protein profiling, to identify nestin as a highly EC-enriched intermediate filament in the human adult, and to characterise its expression in malignancy and atherosclerosis. We demonstrate that the subcellular distribution of nestin is regulated by shear forces and that it has a role in the inhibition of EC proliferation.

We identified nestin as EC-enriched throughout all adult vascular beds. The turnover of adult EC is over months or years^56^ and it is estimated that 99% of the adult vasculature is quiescent^35^, consistent with our observation that EC were negative for markers of proliferation. However, current dogma dictates that nestin is a neovascularisation marker, absent from non-proliferating adult EC^3,18,19,57,58^; a concept underpinned by studies showing angiogenic EC in the human embryo and corpus luteum were nestin positive^40^, whilst nestin was largely absent from the vasculature of the adult rat^57^ and mouse^19^. However, until now there has been no systematic study of nestin expression in the human adult, although there are isolated reports of nestin-positive EC being detected in adult human brain^59^ and pancreas^60^. There is divergence between the sequence and promoter regions of the *NES* gene between species^61,62^, which could account for the discrepancies between our observations and those in animal models^19,57^; indeed, it is well documented that key EC genes can vary between rodents and humans^63–65^.

We found no evidence that nestin expression profiles in kidney, bladder, lung and stomach tumours were distinct from established EC markers. Previous reports suggest nestin is a specific marker for angiogenic EC in various tumour types, and could be useful as a therapeutic target^18,21,22,40,58,66^. We did find that higher nestin expression was associated with unfavourable kidney cancer prognosis, consistent with previous studies^66^. However, we found a similar association with PECAM1 and CD34, suggesting that these EC genes represent a proxy for the degree of vascularisation, a factor previously linked with outcome^67^. Nestin expression was markedly different in glioblastoma (GBM) compared to the other tumour types, with high levels of expression outside the vasculature; as previously reported^20,68,69^. As GBM cell subpopulations can differentiate into EC-like cells (‘vascular mimicry’)^70,71^, it would be interesting to consider the role of nestin, as an EC-specific IF, in this process.

Genes with highly EC restricted expression across tissue beds tend to be important for vascular stability or EC-specific functions^72–74^. We demonstrated that nestin subcellular spatial arrangement is subject to shear stress regulation in primary EC from multiple vascular beds. Such redistribution, from a perinuclear aggregate to a filamentous network, is characteristic of IF polymerisation. IF polymerisation status is associated with exposure to stimulus such as hypoxia^75^, mechanical, chemical or toxin induced stress^76–79^ and changes in phosphorylation^76–78,80^. Nestin has many potential phosphorylation sites^81^, the most well studied associated signalling pathway being the regulation of the kinase CDK5 and its co-activator p35^16,17,80,82^. Although this signalling pathway has not been studied in EC, CDK5 has previously been linked to the regulation of EC migration and angiogenesis^83^. We observed subcellular co-localisation between EC nestin and vimentin under static conditions and following shear stress induced redistribution, however the subcellular distribution of vimentin did not appear to be disrupted by nestin inhibition. Vimentin is the most well studied EC IF to date but, unlike nestin, it is not highly EC-specific in the adult^49,50,84^. Vimentin is important for EC-specialised functions, such as lymphocyte recruitment^85^, basement membrane interactions^86^ and the formation of Von Willebrand factor strings on the EC surface^87^. The functional relevance of nestin-vimentin co-localisation in such processes is currently unknown.

We found that silencing of the *NES* gene in *in vitro* increased EC proliferation, but did not affect migration. Previous studies also reported that *NES* silencing had no effect on EC migration^21^, but reported inhibition of EC proliferation^21,88^. Experimental differences could contribute to this apparent discrepancy, including the use of immortalised murine and human EC lines^21^, in contrast to human primary EC, and the use of VEGF as a proliferation stimulus, following serum starvation^88^, compared to the measurement of spontaneous replication. *NES* silencing can also increase proliferation capacity in terminally differentiated human podocytes^32^, indicating that nestin may also influence cell cycle progression in other cell types where it is constitutively expressed.

We found a reduced expression of *NES* mRNA in human carotid atherosclerotic plaques, compared to normal arteries, corroborated by reduced or absent neovessel EC nestin protein expression. The role of nestin in human atherosclerosis is not well studied, although the disease-associated modified shear stress is well known to affect several processes in which we have here demonstrated a functional role of nestin, such as cytoskeletal rearrangement, wound repair and proliferation^89,90^. We identified a haplotype associated with reduced *NES* mRNA expression in aortic EC under basal conditions. This haplotype includes two genetic variants previously linked to early onset CAD^54^, but the connection with cardiovascular risk remains an open question, especially as no genetic variation at the *NES* locus had emerged in large genome-wide genetic association studies for such diseases.

Our study challenges the current dogma that nestin is restricted to areas of tissue regeneration in the adult, showing that it is an integral body-wide EC-enriched protein. Thus, nestin is not a suitable marker for human adult progenitor/stem cells, or therapeutic target to inhibit neovascularisation, due to its widespread EC expression. Examining the transcriptional networks controlling nestin expression in EC vs. other cell types during development could provide new insights into its role in development, EC function and pathology.

## Materials and Methods

### Experimental approval

All methods were carried out in accordance with relevant guidelines and regulations and all experimental protocols were approved by the relevant institutional committee. Further specific details are provided in each relevant section below, and/or appropriate reference provided.

### Analysis of human normal and cancer tissues

Samples of normal and cancer tissues used for mRNA and protein expression analysis were obtained from the Department of Pathology, Uppsala University Hospital, Uppsala, Sweden; as part of the Uppsala Biobank. Samples were handled in accordance with Swedish laws and regulations, in accordance with approval and advisory report from the Uppsala Ethical Review Board, as previously described^27,44^.

### Transcript Profiling (RNA-seq)

#### Normal tissue

Human tissue transcript profiling was performed in house as part of the Human Protein Atlas (HPA) project (www.proteinatlas.org)^27^. 176 individual human tissue samples were collected from 37 different organs (details in Table S1). Tissue samples were embedded in optimal cutting temperature compound and stored at −80 °C. Hematoxylin and eosin (HE) stained frozen sections (4 μm) were prepared from each sample and examined by a pathologist to confirm sampling of representative normal tissue. Three sections per sample were homogenised using a 3 mm metal grinding ball (VWR) and total RNA was extracted using the RNeasy Mini Kit (Qiagen), according to the manufacturer’s instructions. Extracted RNA was analysed using either an Experion automated electrophoresis system (BioRad Laboratories) with the standard-sensitivity RNA chip or an Agilent 2100 Bio-analyser system (Agilent Biotechnologies) with the RNA 6000 Nano Labchip Kit. Only high quality RNA (RNA integrity number ≥7.5) was used for library preparation (PolyA) and sequencing. Next generation RNA sequencing was performed using Illumina Hiseq2000 and Hiseq2500 and the standard Illumina RNA-seq protocol with a paired end read length of 100 × 2 bp or 125 × 2 bp with on average 50 M reads/library (span of 13–84 M reads). Processed reads were mapped to the Human Genome (GRCh37 and GRCH38) using Tophat v2.0.8b^91^, allowing for two mismatches. Transcript abundance FPKM (fragments per kilobase of exon model per million mapped reads) values were calculated using Cufflinks v2.1.2^92^ and Ensembl build 75^93^ or Ensembl build 83^94^ using summarised gene TPM, not accounting for different isoforms in the analysis. The number of protein coding genes mapped was 20,344.

#### Tumour tissue

Expression levels of *PECAM1*, *VWF*, *CD34* and *NES* transcripts in kidney renal clear cell carcinoma (KIRC, n = 178), bladder urothelial carcinoma (BLCA, n = 132), lung adenocarcinoma (LUAD, n = 191), stomach adenocarcinoma (STAD, n = 215) and glioblastoma multiforme (GBM, n = 166), and corresponding normal tissue (n = 72, 19, 59, 35, 5, respectively) were collected from The Cancer Genome Atlas (TCGA) (https://cancergenome.nih.gov/) and Genotype-Tissue Expression (GTEx) project (https://www.gtexportal.org/home/), though the Firebrowse (http://firebrowse.org/) or OASIS portal (http://www.oasis-genomics.org/)^95^.

### Protein profiling: Normal and tumour tissue

Tissue microarrays (TMA) were generated and stained as part of the HPA project, as previously described^27^. Briefly, formalin fixed and paraffin embedded tissue samples were sectioned, de-paraffinised in xylene, hydrated in graded alcohols and blocked for endogenous peroxidase in 0.3% hydrogen peroxide diluted in 95% ethanol. For antigen retrieval, a Decloaking chamber® (Biocare Medical, CA) was used. Slides were boiled in Citrate buffer®, pH6 (Lab Vision, CA). Primary antibody against NES (HPA007007), CLEC14A (HPA039468), VWF (HPA001815), CD34 (HPA036722), PECAM1 (HPA004690), PCNA (HPA030522), MKI67 (HPA001164) (all Atlas Antibodies) and CDK2 (AHZ0142, BioSource) and a dextran polymer visualization system (UltraVision LP HRP polymer®, Lab Vision) were incubated for 30 min each at room temperature and slides were developed for 10 minutes using Diaminobenzidine (Lab Vision) as the chromogen. Slides were counterstained in Mayers hematoxylin (Histolab) and scanned using Scanscope XT (Aperio).

### Human cancer patient (survival) data

Cancer patient samples used for mRNA expression and survival analysis were collected from The Cancer Genome Atlas (TCGA) project from the initial release of Genomic Data Commons (GDC) on June 6, 2016, as previously described^96^ and with extended information found at https://portal.gdc.cancer.gov/. The samples used in the study included only those with both clinical information and transcriptomic data available at that time point.

### Primary endothelial cell culture and treatments

Ethical approval for endothelial cell isolation and subsequent experimentation was granted by *Regionala etikprövningsnämnden i Stockholm* (diarienummer 2015/1294-31/2). Human Umbilical Vein Endothelial Cells (HUVEC) were isolated as previously described^97^, from anonymised umbilical cords collected from Karolinska University Hospital. HUVEC were grown in Medium 199 (M199, Gibco) supplemented with 20% foetal bovine serum (FBS), 100 U/ml penicillin, 0.1 mg/ml streptomycin, 1 μg/ml Hydrocortisone, 1 ng/ml Human Epidermal Growth Factor (all Sigma), and 1.25 μg/ml Amphotericin B (Invitrogen). Human Pulmonary Artery Endothelial Cells (HPAEC), Human Coronary Artery Endothelial Cells (HCAEC), and Human Dermal Microvascular Endothelial Cells (HDMEC) were obtained from Promocell in cryogenically frozen vials, and were cultured in EC Growth Medium (HPAEC, HCAEC) or EC Growth Medium-MV (HDMEC) (Promocell). For some experiments, serum starvation was carried out using 2% or 0.5% FBS.

### siRNA transfection

Endothelial cells were treated with two different siRNA sequences targeting nestin (s21141, s21142, Ambion) or a scrambled control (4390843, Ambion) using Lipofectamine RNAiMAX transfection reagent (Invitrogen) according to manufacturer instructions. 72 hours after transfection, nestin mRNA expression was inhibited by >75% compared to untransfected cells (Fig. S3A), and antibody staining of nestin protein was greatly reduced (Fig. S3B,C).

### Laminar shear stress

Endothelial cells were cultured to confluence in plastic flow chamber slides (μ-slide VI^0.4^, Ibidi GmbH). The slide was then attached to an Ibidi fluidic unit and pump system using silicone tubing, and a continuous unidirectional laminar shear stress of 10 dyne/cm^2^ was applied for 24 hours.

### Immunofluorescence staining

EC were fixed using 4% paraformaldehyde solution in PBS, permeabilised in 0.5% triton X-100 and then blocked using 5% BSA. Primary antibody against nestin (HPA007007, Atlas Antibodies), Vimentin (OMA1-06001, Invitrogen) or PCNA (LifeSpan Biosciences), was incubated with the cells for 20 minutes, followed by FITC-conjugated anti-rabbit antibody (F-9887, Sigma), or Alexa-Fluor 647 conjugated anti-mouse antibody (A-21235, Invitrogen). Some experiments were also actin-stained with TRITC-conjugated phalloidin (P1951, Sigma). Cells were covered with 40 μl DAPI-containing mounting medium (Vectashield) before imaging. Fluorescence images were taken using a Leica TPS SP5 confocal microscope, and were processed using Fiji image processing software^98^. For measurement of percentage of nestin coverage, between 10–30 cells per image were individually selected and outlines saved as separate overlays using selection tools and the Region of Interest (ROI) manager, and coverage determined using the default threshold tool. Cell area was determined using actin staining and a high contrast filter/look up table. Co-localisation was calculated for each individual cell using the Coloc2 plugin.

### qPCR

cDNA was prepared using TaqMan Gene Expression Cells-to-Ct Kit (Ambion), and qPCR was subsequently performed using Taqman Fast Universal PCR Master Mix and 18 s rRNA reference primer (4319413E), with target primers for nestin (Hs04187831_g1) and vimentin (Hs00958111_m1) using a StepOnePlus Real-Time PCR System (all Applied Biosystems).

### Gap closing assay

Cells were seeded into 24 well plates, 24 hours after siRNA treatment. The following day, cells were washed, and a gap was created by carefully scratching a 100 μl pipette tip across the centre of the well. Cells were washed in PBS, and medium replaced with either normal (20% FCS), or serum starvation (0.5% FCS) medium. The plate was placed onto a Nikon Ti-E phase contrast microscope stage, housed inside a 37 °C, 5% CO_2_ fed chamber, and photographs of each scratch were taken once each hour for 96 hours using NIS Elements image capture software. Images were exported into Fiji for image processing, and the size of the gap measured by area every 12 hours.

### Cell migration and cell division assay

Cells were seeded at 60% confluence to 12 well plates, before treatment with siRNA. After siRNA treatment, cells were washed and fed with medium, and then placed on a Nikon Ti-E phase contrast microscope stage inside a 37 °C, 5% CO_2_ fed chamber. Photographs of each well were taken once each hour for a period of 96 hours using NIS Elements image capture software, and then exported into Fiji for image processing, cell counting and migration analysis. The number of cells in each field of view every 24 h was counted manually using the Cell Counter plugin, and the increase in cell number since 0 h calculated. Migration was measured using the Manual Tracking plugin. The positions of 20–30 individual cells per image were tracked over 96 h, and mean migration velocity calculated for each 24 h period.

### Plaque microarray and staining

Microarray analysis and plaque staining was carried out as part of the bank of Karolinska Endarterectomy (BiKE), as previously described^53^. Briefly, patients undergoing surgery for high-grade (>50% NASCET) carotid stenosis at the Department of Vascular Surgery, Karolinska University Hospital, Stockholm, Sweden were consecutively enrolled in the study, clinical data recorded on admission and carotid endarterectomies (carotid plaques) collected at surgery. For microarrays, plaques were divided transversally at the most stenotic part, the proximal half of the lesion used for RNA preparation while the distal half was processed for histology. Normal artery controls (NA) were nine macroscopically disease-free iliac arteries and one aorta, obtained from organ donors without history of cardiovascular disease. All samples were collected with informed consent from patients or organ donors’ guardians. The BiKE study is approved by the Ethical Committee of Northern Stockholm with following ethical permits: EPN DNr 95-276/277; 02-146; 02-147, 2005/83-31; 2009/512-31/2; 2009/295-31/2; 2011/950-32; 2012/619-32 and 213/2137-32. The project is performed under the Swedish biobank regulations and prospective sampling is approved with informed consent procedure (DNr 2009/512-31/2). BiKE is registered at Socialstyrelsen (The National Board of Health and Welfare) and Biobank of Karolinska and approved by the Swedish Data Inspection Agency (approval date/number 2002-09-30 DNr 916-2002). The BiKE database was merged with the Swedish Hospital Discharge Register and the Swedish Cause of Death Register for follow-up of major adverse cardiovascular, cerebrovascular and vascular events (MACCEs). All samples were collected with informed consent from patients or organ donor guardians. The BiKE microarray dataset is available from Gene Expression Omnibus (GSE21545).

For immunohistochemistry, all reagents were purchased from Biocare Medical (Concord, CA). Tissues were fixed in 4% Zn-formaldehyde for 48 hours, dehydrated in 70% ethanol and embedded in paraffin blocks. A probe-polymer system containing alkaline phosphatase (AP) and horseradish peroxidase (HRP) was applied, with subsequent detection of NES (HPA007007, Atlas Antibodies) using Warp Red and VWF (#M0616, DAKO) using Vina Green chromogenes. Slides were counterstained with Hematoxylin QS (Vector Laboratories, Burlingame, CA), dehydrated and mounted in Pertex (Histolab, Gothenburg, Sweden). Images were taken using an automated SlideScanner System.

### eQTL analysis

Expression quantitative locus (eQTL) mapping of EC gene expression has been described^55^. Briefly, participants were genotyped using the Affymetrix Genome-Wide Human SNP Array 6.0. Gene expression profiles were determined using the Affymetrix HT HG-U133A microarray. Single nucleotide polymorphisms with genotyping call rate <95%, minor allele frequency (MAF) < 0.01 or showing signicant (p < 10^−6^) deviation from Hardy-Weinberg equilibrium were filtered out. This led to 799,085 quality-control (QC) validated autosomal SNPs and 147 individuals. Genotype data were then imputed using the 1000 Genomes phase 3 reference dataset with the MACH (version 1.0.18) software. Associations between imputed genotypes and *NES* expression were computed using a linear model regression where the imputed allele dosage was used as covariate to assess SNP’s effect. Imputed SNPs with imputation quality criteria greater than 0.3 were kept for analyses. Analyses were conducted by use of the Matrix_eQTL_main function (MatrixEQTL R package) while adjusting for the main principal components derived from genetic data. eQTL effects were considered as cis (local) when the SNP was located within a 10^6^ bp distance upstream or downstream from *NES* probe sequence.

### Software

Image analysis was carried out using Fiji image processing software^98^ and NDP.view2 (Hamamatsu), graphs and calculations used Graphpad Prism 7 and Microsoft Excel. Figures were assembled using Adobe Photoshop.

## Electronic supplementary material

Supplemental marked for review

Supplemental unmarked

Supplemental Table 2

Supplemental Table 3

## Data Availability

Human tissue sequencing data is available in our previous publication^27^ and deposited in ArrayExpress under accession number E-MTAB-2836. The Human Protein Atlas (HPA) website contains details of all sequencing data and antibody-based protein profiling used in this study: www.proteinatlas.org.
