# Supplementary material for: A systems-approach reveals human nestin is an endothelial-enriched, angiogenesis-independent intermediate filament protein
Source: Sci Rep. 2018 Oct 2;8:14668. doi: 10.1038/s41598-018-32859-4 (PMC6168570; doi:10.1038/s41598-018-32859-4)
Supplement: Supplementary file 1 — Supplemental marked for review [file 41598_2018_32859_MOESM1_ESM.docx]

##
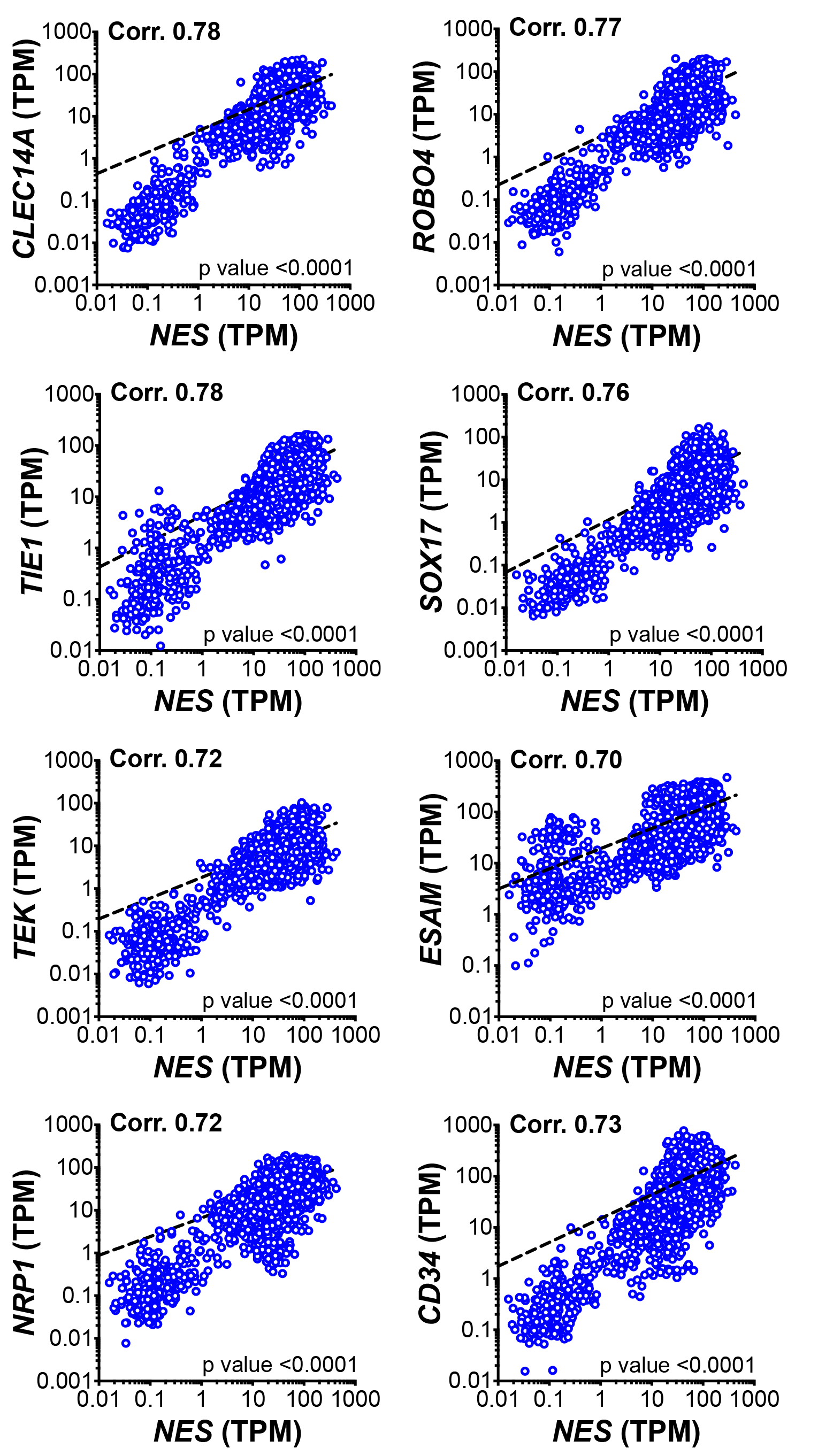


##
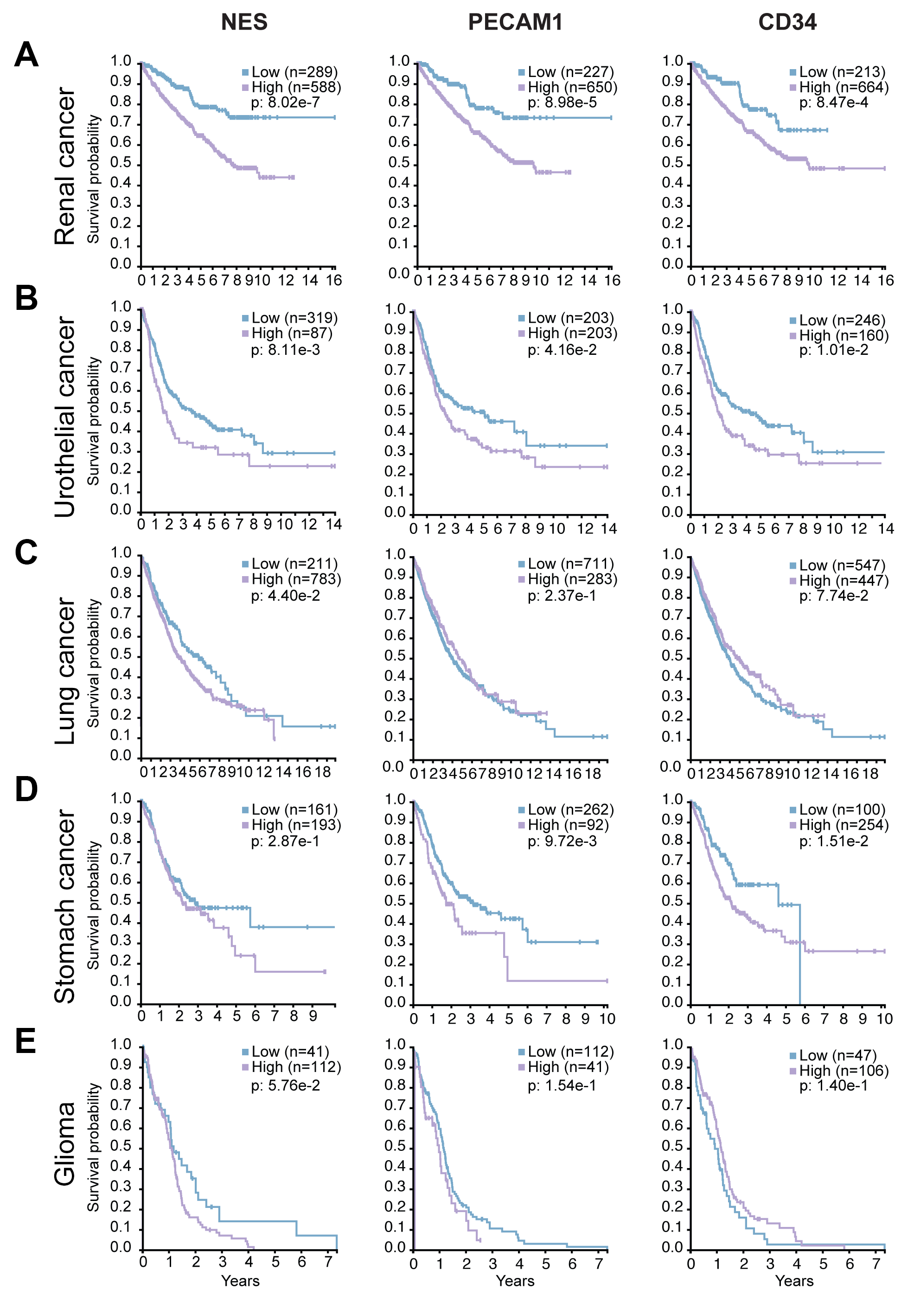


##
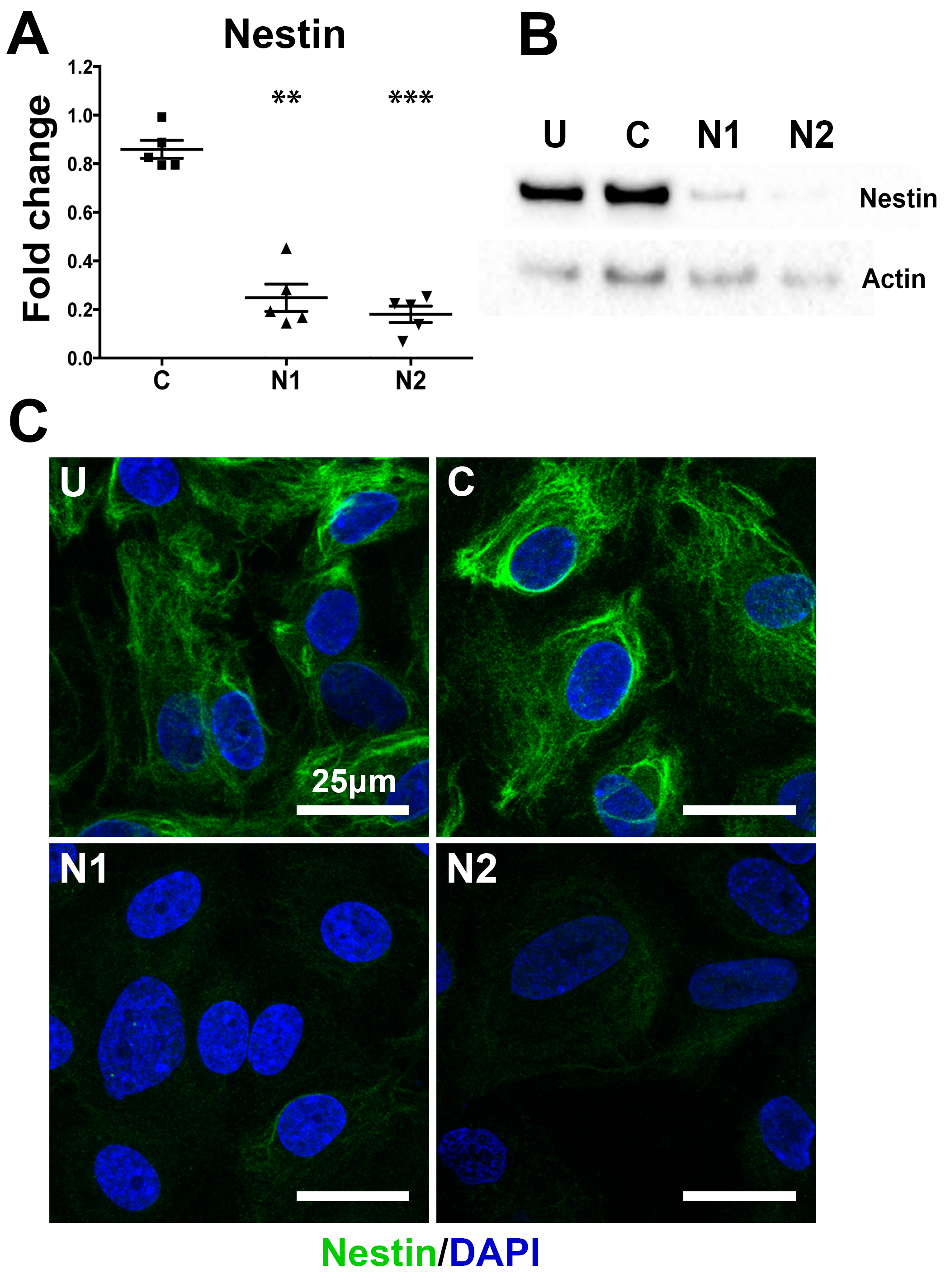


##
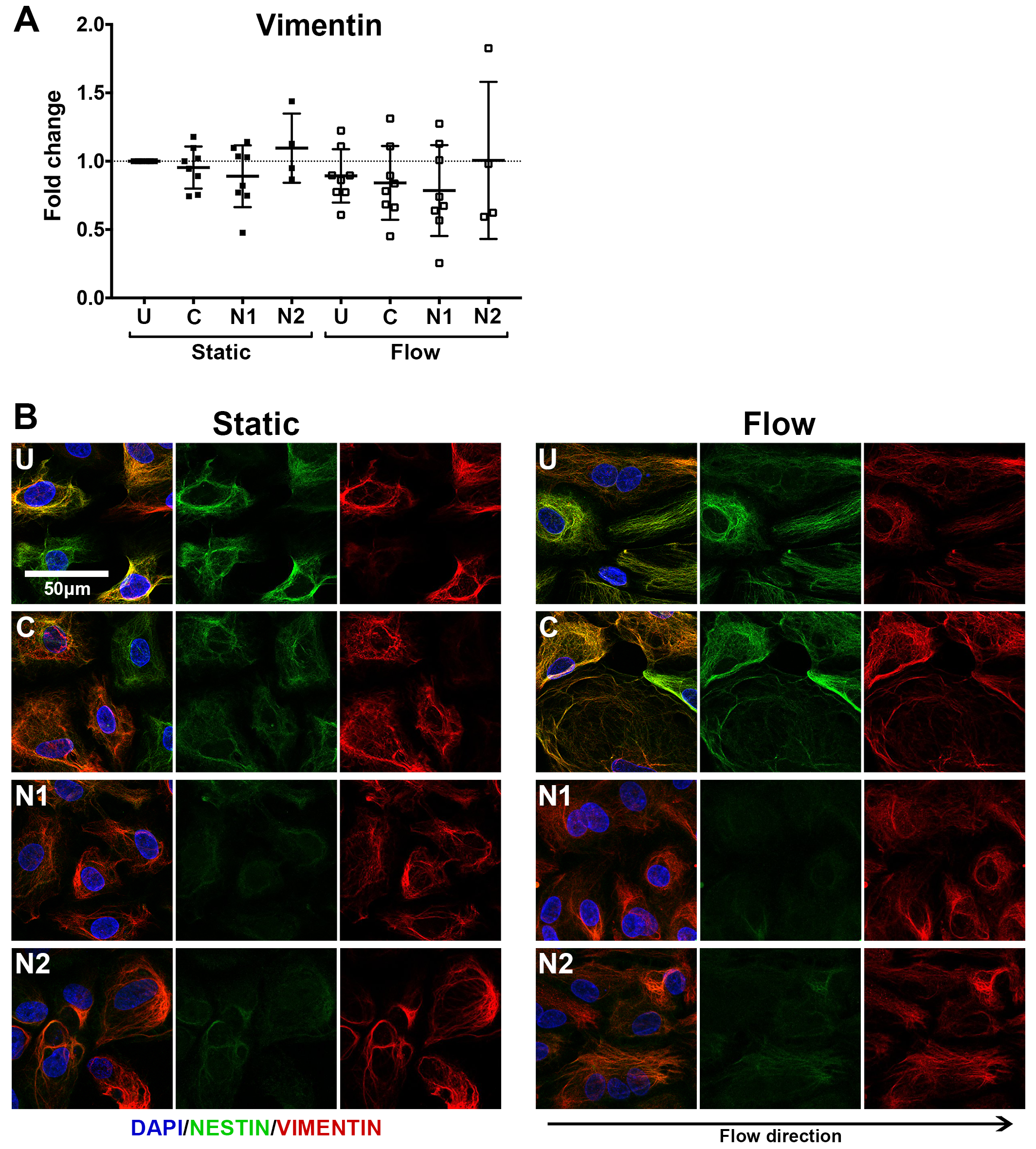


## Supplemental Figure Legends

## Figure S1: EC marker transcripts values correlate with *NES* in GTex dataset: Related to Figure 1. RNA-seq data from 2841 individual samples from 25 different human tissues from the GTex project were used to generate Spearman pair wise correlation values between *NES* and those encoding for the known EC transcripts *CLEC14A*, *ROBO4*, *TIE1*, *SOX17*, *TEK*, *ESAM*, *NRP1* and *CD34.* Correlation values and corresponding p-values are shown in the top left and bottom right of each scatter plot, respectively.

## Figure S2: Correlation between mRNA expression level and patient survival: Related to Figure 3. Kaplan-Meier plots summarise results from analysis of correlation between mRNA expression level and patient survival, using best separation, for the genes *NES*, *PECAM1* and *CD34* in (A) renal cancer, (B) urothelial cancer, (C) lung cancer, (D) stomach cancer, and (E) glioma. Patients were divided, based on expression level, into a ‘low’ or ‘high’ group. For each Kaplan-Meier plot, corresponding 5-year survival for patients with high expression, 5-year survival for patients with low expression and log-rank P value are displayed. For glioma, 3-year survival is shown. All three genes show significant (p<0.001) association with patient survival in renal cancer with unfavourable prognosis. The survival analysis is described in more detail at the HPA portal ([www.proteinatlas.org/about](https://email.ki.se/owa/redir.aspx?C=7CXJrQpb6Kxe3EhXSYcsxrcd2zbXSQhODXvPrM8DtSlq1MM_227VCA..&URL=http%3A%2F%2Fwww.proteinatlas.org%2Fabout%2Fassays%2Bannotation%23tcga_survival)).

## Figure S3: Verification of nestin antibody specificity and knockdown efficiency: Related to all Figures. HUVEC were transfected with control siRNA, or one of two different anti-nestin siRNA sequences. (A) *NES* mRNA expression 72h post-transfection measured by qPCR, and nestin protein expression analysed by (B) Western blot and (C) immunofluorescence staining. (U-Untransfected cells, C-Scrambled control siRNA, N1/N2-anti-*NES* siRNA).

## Figure S4: Vimentin expression and subcellular organisation is not affected by nestin inhibition. HUVEC were untreated (U) or transfected with control (‘C’) or one of 2 anti-nestin (‘N1’ and ‘N2’) siRNAs and cultured under static conditions, or 10dyne/cm^2^ laminar shear stress (‘flow’) for 24 hours, before measurement of (A) *VIM* mRNA expression and (B) subcellular organisation of nestin and vimentin protein by immunofluorescence staining. Graph shows means ±SD.

##
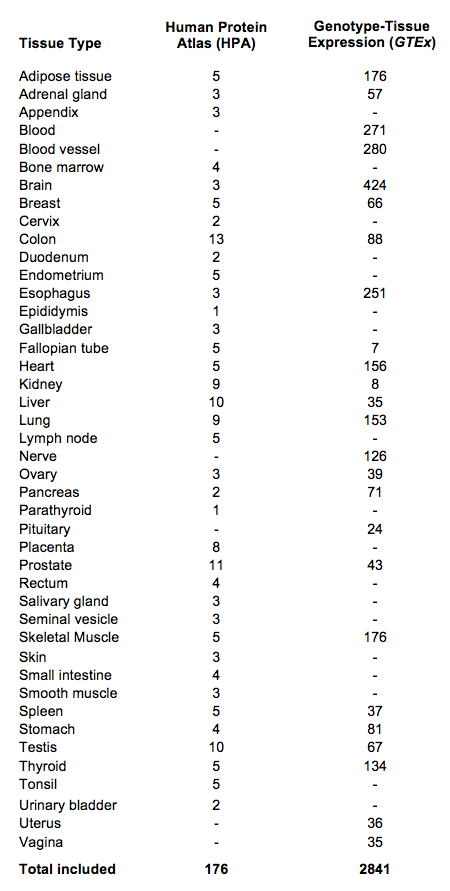


## Table S1: Source and number of human tissue samples in the HPA and GTEx RNAseq dataset: Related to Figure 1 and 2. 176 individual samples from 37 different organs (HPA) and 2841 different samples from 25 different organs (GTex) were analysed for RNA-seq.

**Supplemental Excel Tables:**

**Table S2: 150 transcripts most highly correlated with *NES* and corresponding gene ontology grouping analysis: related to Figure 1.** RNA-seq data from 176 individual samples from 37 different human tissues were used to generate Spearman pair wise correlation values between *NES* transcript values and those encoding for all other mapped protein coding genes. Column **A-B**: Gene ID and Spearman pair wise correlation values for the 150 transcripts whose expression most highly correlated with *NES,* across all tissues. Column **D-K:** Gene ontology analysis of these 150 transcripts, using PANTER (<http://geneontology.org/>), GO ontology database release date 2018-02-02) to identify over or under represented biological process. Grey shaded text indicates a false discovery rate (FDR) >0.001. Terms with FDR >0.01 are not displayed.

**Table S3: Tab 1:** Expression quantitative trait (eQTL) analysis of the NES gene locus using GWAS and RNA expression data from cultured aortic ECs (HAEC) from 147 individuals. **Tab 2:** Haplotype analysis of the rs3748570, rs11582300, and rs3935541 SNPs on *NES* expression.
